# A case series of low dose bevacizumab and chemotherapy in heavily pretreated patients with epithelial ovarian cancer

**DOI:** 10.1186/1757-2215-5-17

**Published:** 2012-06-25

**Authors:** Carlotta Defferrari, Sara Campora, Mauro D'Amico, Arnoldo Piccardo, Ennio Biscaldi, Daniela Rosselli, Ambra Pasa, Matteo Puntoni, Alberto Gozza, Alessandra Gennari, Silvia Zanardi, Rita Lionetto, Michela Bandelloni, Andrea DeCensi

**Affiliations:** 1 Unit of Medical Oncology, E.O. Ospedali Galliera, Mura delle Cappuccine 14, 16128, Genoa, Italy; 2 Nuclear Medicine, E.O. Ospedali Galliera, Genoa, Italy; 3 Radiology, E.O. Ospedali Galliera, Genoa, Italy; 4 Gynecology, E.O. Ospedali Galliera, Genoa, Italy; 5 Scientific Direction, E.O. Ospedali Galliera, Genoa, Italy; 6 Health Direction, E.O. Ospedali Galliera, Genoa, Italy; 7 Pharmacy, E.O. Ospedali Galliera, Genoa, Italy

## Abstract

**Background:**

The addition of bevacizumab to standard chemotherapy prolongs progression free survival in the first line treatment of epithelial ovarian cancer (EOC), but its cost/effectiveness is debated. We assessed the safety and activity of a lower dose of bevacizumab in pretreated advanced stage EOC.

**Methods:**

We treated 15 patients, mostly with platinum resistant EOC, who had received a median of four prior cytotoxic regimens, with bevacizumab 5–7.5 mg/kg q21 days in combination with either carboplatin (n = 8), oral cyclofosfamide (n = 5) or weekly paclitaxel (n = 2). Bevacizumab was administered until disease progression. Tumor response was assessed by CA125 and fusion ^18^ F-FDG PET/contrast enhanced CT.

**Results:**

The median number of bevacizumab cycles was 21 (range 3–59). The median baseline CA125 was 272 U/ml and decreased to 15.2 U/ml at nadir. Tumor response was 4 complete response (CR) (26.7%) and 7 partial response (PR) (46.7%) by chemotherapy (CT), with an overall response rate of 73.4% (95% CI, 51.0 – 95.8) according to Response Evaluation Criteria In Solid Tumors (RECIST), and 6 CR (40%) and 4 PR (26.7%) by PET, for an overall metabolic response rate of 67% (95%CI, 42.8 – 90.6) according to PET Response Criteria in Solid Tumors (PERCIST). Median progression free survival (PFS) was 21 months and median overall survival (OS) was 24 months. Grade 3 adverse events related to bevacizumab were hypertension (n = 2), proteinuria (n = 1) and epistaxis (n = 5). Treatment was delayed in five patients for nasal bleeding or uncontrolled hypertension.

**Conclusions:**

Low-dose bevacizumab and chemotherapy was well tolerated and active in a heavily pretreated population of advanced EOC. Further studies should assess the activity of low dose bevacizumab in EOC.

## Introduction

Ovarian cancer is the most lethal gynecologic malignant tumor in developed countries. It is generally diagnosed in advanced stage and cytotoxic chemotherapy is only partially effective. The doublet carboplatin and paclitaxel has been the standard of care for more than 15 years, and all attempts to add new agents have so far been unsuccessful.

Recently new targeted treatments have been explored in epithelial ovarian cancer (EOC), with angiogenesis being one of the most studied targets for inhibition of tumor growth and metastasis [1,2]. Among these targets, the vascular endotelial growth factor (VEGF) family consists of seven glycoproteins involved in tumor angiogenesis [3]. Overexpression of VEGF is often observed in solid tumors and has been demostrated in ovarian cancer, and a correlation with poor prognosis and increased risk of metastatic disease has been noted [4,5]. The VEGF pathways have therefore been regarded as a promising target to treat EOC [6].

Bevacizumab (Avastin®) is a humanized monoclonal antibody that target VEGF-A and has been registered for the treatment of advanced disease of colon, non-small-cell lung, breast, renal and refractory glioblastoma. A number of phase-II trials have assessed the activity of bevacizumab in the treatment of recurrent EOC, with promising results [7,8], including patients with platinum resistant disease [9-11]. These trials were followed by three randomized phase-3 trial in first-line treatment of EOC: GOG 218, ICON 7 and OCEAN trial [12-14].

Results of these trials have shown significant prolongation of progression free survival (PFS) with the addition of bevacizumab to standard doublet chemotherapy, with ICON 7 showing also a favorable trend in overall survival (OS), based on which the European Medicines Agency (EMA) has recently approved its use in EOC on 4^th^ October 2011 [15]. In these randomized trials toxicity was not negligible, the most common adverse events being G3-4 hypertension in 3–14.8%, dose-limiting proteinuria in 0.8%-4%, hemorrhage and thrombotic events up to 3%, impaired would healing in 1–3.7% and gastrointestinal perforation in 0%-1.5% of the patients [16].

Treatment related toxicity and financial costs represent an important issue for expensive drugs like bevacizumab. Cohn et al. [17] have recently analyzed the cost effectiveness of bevacizumab in GOG 218 trial, where the dose of bevacizumab was 15 mg/kg q21 and concluded that the addition of bevacizumab to the adjuvant treatment was not cost effective. The maintenance treatment improved PFS, but direct and indirect costs raised substantially [17]. These considerations, coupled with the results of ICON 7 trial which showed an improved PFS in patients receiving bevacizumab 7.5 mg/kg q21, prompted us to explore the activity of low dose bevacizumab in advanced EOC. Notably, only a few dose-finding studies [18] in cancer patients have so far been conducted, mostly with inconclusive findings, so that the optimal biological dose of bevacizumab in most advanced neoplasms, including EOC, remains unclear. In contrast, several dose ranging trials have been conducted in the treatment of macular degeneration, with equivalent efficacy but fewer toxicity of the lower doses [19,20]. We explored the activity and safety of 5–7.5 mg/kg q21 bevacizumab in heavily pretreated patients mostly with platinum resistant EOC.

### Patients and methods

This was a mono-institutional case-series performed under Institutional Review Board support using an off-label indication of bevacizumab in heavily pretreated patients with EOC. Our hospital operating procedure for off label indication was used to treat such patients according to the Italian law. Informed consents were collected and declarations of responsibility was signed by the Chief of the Unit of Medical Oncology for all patients entered in this study. Eligibility criteria included at least three prior treatment lines, presence of measurable disease, ECOG performance status 0–1, life expectancy of at least 3 months, adequate bone marrow, renal and liver function. Exclusion criteria included bleeding or clotting disorders, prior or current significant cardiovascular disease, including uncontrolled hypertension. Treatment consisted of bevacizumab 5 mg/kg q21 in 10 patients and 7.5 mg/kg q21 in 5 patients in combination with carboplatin AUC2 weekly (n = 2) or AUC5 q21 (n = 6) or oral cyclofosfamide 50 mg/day (n = 5) or weekly paclitaxel (n = 2). Ten patients with body mass index (BMI) <25 received 5 mg/kg, whereas five patients with BMI ≥25 received 7.5 mg/kg. This adjustment was done to minimize the risk of renal toxicity. The choice between oral and intravenous CT was based on patient’s preferences and logistical considerations. The two patients who receive paclitaxel had developed platinum intolerance. Bevacizumab was administered until disease progression or unmanageable adverse event. Dose reduction of bevacizumab was not permitted but was withheld for any grade 3 or greater bevacizumab-related toxicity until recovery to grade 1. Chemotherapy was withheld and reduced by 20% for grade 4 neutropenia, thrombocytopenia, grade 3 or greater hepatic toxicities, grade 2 or greater renal toxicity or other toxicity impacting organ function. The use of granulocyte colony stimulating factors and erytrocyte stimulating factors was at physician discretion.

Data extracts included demographics, baseline clinical data, adverse events, and response to treatment. Safety was evaluated with tests to detect known toxicities of bevacizumab as high blood pressure, proteinuria by urinary protein monitoring and close monitoring of GI toxicity. The NCI CTCAEv4.0 criteria were used.

Each patient underwent integrated ^18^ F-FDG PET/Contrast enhanced (Ce)CT before starting the study, every 3 months and when disease progression was suspected by increasing levels of CA125. ^18^ F-FDG PET/CT and CeCT were acquired at the same time using the same dedicated PET/CT scanner (Discovery LS, GE Medical Systems, Milwaukee, WI, USA) and the integrated ^18^ F-FDG PET/CeCT protocol was standardized as previously described [21].

Briefly, a conventional whole-body PET/CT was performed with 50' after injection of 370 MBq of ^18^ F-FDG; colon distension was performed inflating 2 L of room air during intestinal pharmacological hypotonization (20 mg of endovenous Joscine bromide), followed immediately by abdominal PET acquisition. Finally, a CeCT of the abdomen (120 KV, 350 mAs, 0.5 s rotation tube), was performed during the portal phase, after injection of i.v. iodinate contrast medium (Iopamidol 370 mg/I 100 ml; dose of 1.5 cc/kg of patient weight, injected mechanically at 3 ml/s). ^18^ F-FDG-PET/CT, CeCT and ^18^ F-FDG-PET CeCT studies were interpreted blindly by two nuclear medicine physicians and one radiologist on Advantage Workstation (AW4.2, General Electric Medical Systems), which allowed PET/CT and PETCeCT fusion and MDCT multiplanar reconstructions.

The patients were evaluated for response by Response Evaluation Criteria In Solid Tumors (RECIST) version 1.1 and PET Response Criteria in Solid Tumors (PERCIST) criteria, as recently described [22]. RECIST criteria are typically related to the target lesion dimension, evaluating treatment response in terms of tumor size. In particular tumor size is determined by transaxial imaging performed most commonly with CT. On the other hand PERCIST are PET based response criteria related to the metabolic activity expressed by 18 F-FDG uptake. In this field standardized uptake value (SUV) and SUL (SUV corrected for lean body mass) are the most important parameters able to evaluate treatment response.

According to PERCIST criteria a complete metabolic response is defined as normalization of all lesions to SUL less than mean liver SUL; partial metabolic response is >30% decrease in SUL peak. (minimum 0.8 unit in SUV decrease); progressive metabolic disease is a >30% increase in SUL peak (minimum 0.8 unit increase) or appearance of new lesions or visible increase in extent of FDG uptake. Stable metabolic disease when all previous metabolic criteria were not met.

### Statistical considerations

As a case series, no specific “a priori” sample size calculation was performed. Data on 15 patients were deemed appropriate to provide an initial report on the activity of low dose bevacizumab in patients with similar clinical characteristics.

The standard summary statistics for continuous variables were mean, standard deviation, median, quartiles, maximum and minimum. The standard summary statistics for discrete variables were count and proportion. Tolerability and safety were summarized by the appropriate standard summary statistics. Confidence intervals of response rates were adopted as a measure of the variability of response rate estimates. PFS and OS) were calculated using Kaplan-Meier cumulative survival estimates defined as the time from the day of study entry to, respectively, the first day of clinical or metabolic or radiological progression, and to the day of death from any cause. In case of no event, times were censored to the day of last follow-up and follow-up times of patients dying before progression were censored at the time of death. All calculations were performed using SPSS version 15 and STATA version 11 softwares.

## Results

**Figure 1:**
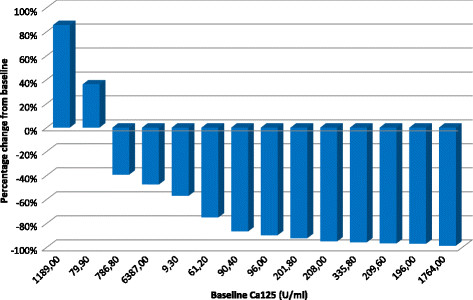
Waterfall plot of best Ca125 response.

Between October 15, 2007 and June 30, 2011, we treated 15 patients with recurrent stage IIIb-IV EOC. The main characteristics of the patients are summarized in Table 1. Briefly, median age was 59 years (range 48–72), median ECOG PS = 1, median BMI 23.1; 13 patients (87%) had serous epithelial histology and 2 (13%) had endometrioid histology. The patients received a median number of four prior cytotoxic regimens (range 3–5). Nine patients (60%) were platinum resistant defined as progression within 6 months after completion of the most recent platinum-based chemotherapy, and six (40%) were partially sensitive. All patients were evaluated for safety and efficacy on July 2011. The median number of bevacizumab cycles was 21 (range 3–59) and the median follow-up time was 19 months (range 5–43). The median baseline CA125 was 272.0 ng/ml and 15.20 ng/ml at nadir. The waterfall plot of the best Ca125 response in each subject is illustrated in Figure 1.

**Table 1:** Main patient characteristics

**ID**	**Age**	**BMI**	**Histologic Subtype**	**Platinum Free Interval**	**Stage**	**Prior Treatment Lines**	**Months of disease before treatment**	**CT Response**	**PET Response**
**01**	56	24	Endometroid	Resistant	III	3	31	Partial Response	Partial Response
**02**	72	19	Serous	Partially sensitive	IV	3	25	Progression Disease	Progression Disease
**03**	59	25	Endometroid	Resistant	IV	3	23	Partial Response	Complete Response
**04**	54	23	Serous	Resistant	IV	4	17	-	Complete Response
**05**	54	21	Serous	Partially sensitive	IV	5	36	Partial Response	Partial Response
**06**	53	26	Serous	Resistant	IV	5	53	Complete Response	Complete Response
**07**	48	17	Serous	Resistant	III	3	26	Partial Response	Partial Response
**08**	56	24	Serous	Resistant	III	3	9	Complete Response	Complete Response
**09**	70	32	Serous	Resistant	IV	5	60	Complete Response	-
**10**	61	28	Serous	Partially sensitive	III	5	68	Partial Response	Progression Disease
**11**	66	26	Serous	Resistant	III	3	28	Partial Response	Complete Response
**12**	70	20	Serous	Partially sensitive	III	3	37	Complete Response	Complete Response
**13**	70	20	Serous	Resistant	III	3	13	Stable Disease	Stable Disease
**14**	49	20	Serous	Partially sensitive	IV	3	27	Partial Response	Partial Response
**15**	62	22	Serous	Resistant	IV	4	8	-	-

**Figure 2:**
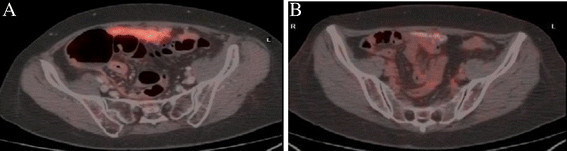
**Peritoneal metastasis from ovarian cancer close to the ascending colon detected by**^**18**^ **F-FDG PET/Contrast enhanced (Ce)CT before starting bevacizumab (A) and at best response (B).**

**Figure 3:**
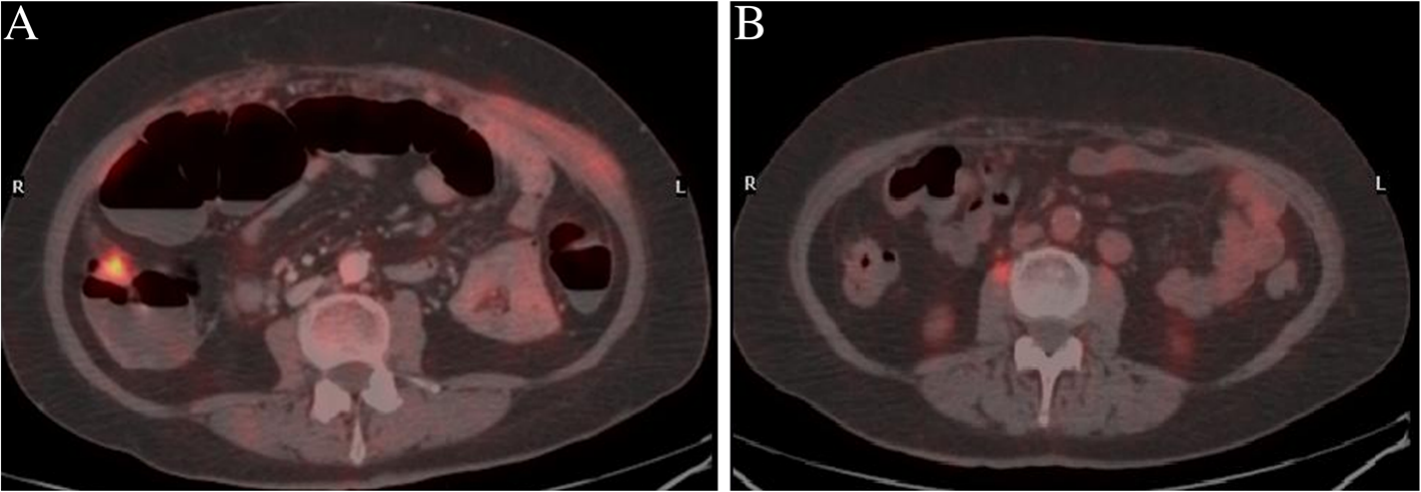
**Peritoneal carcinomatosis from ovarian cancer detected by**^**18**^ **F-FDG PET/Contrast enhanced (Ce)CT before starting bevacizumab (A) and at best response (B).**

Tumor response according to RECIST and PERCIST criteria is summarized in Table 1. According to RECIST a complete response (CR) was observed in 4 patients (26.7%) and a partial response (PR) in 7 patients (46.7%), with an overall response rate of 73.4% (95% CI, 51.0 – 95.8). According to PERCIST criteria 6 patients (40%) had a CR and 4 (26.7%) had a PR, with an overall response rate of 66.7% (95% CI, 42.8 – 90.6). Four of the nine platinum resistant patients were treated with carboplatin + bevacizumab: three achieved a partial response and one had a complete response. Two cases of complete morphological and metabolic response are illustrated in Figures 2 and 3.

**Figure 4:**
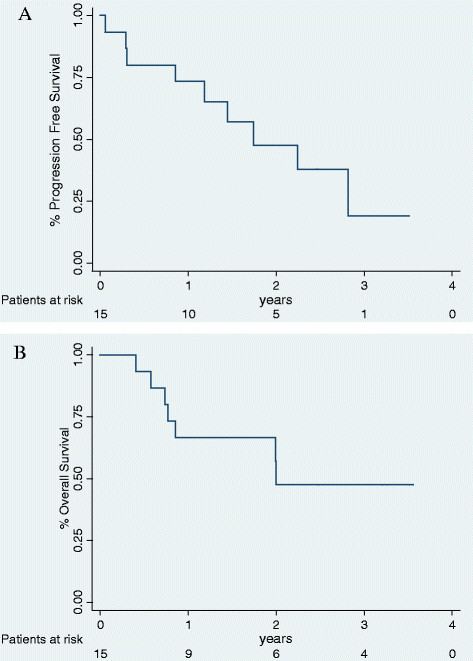
Kaplan-Meier progression-free survival (A, top panel) and overall survival (B, bottom panel).

Median PFS was 21 months (interquartile range, 10–34) and median OS was 24 months (Figure 4).

The majority of adverse events were mild or moderate (proteinuria n = 5, hypertension n = 8). Grade 3 adverse events related to bevacizumab were hypertension (n = 2) and proteinuria (n = 1). Epistaxis was noted in 5 cases but no treatment was required. The treatment was never stopped for toxicity, but was delayed in five patients for bleeding (n = 2) and uncontrolled hypertension (n = 3). Seven patients complained of G1 abdominal pain. There were no cases of bowel perforation or grade 4 toxicity.

## Discussion

The standard of care in recurrent, platinum resistant EOC after three lines of chemotherapy remains unclear since no chemotherapy agent has ever demonstrated superior activity over another. Topotecan is usually employed in this setting but a low response rate and its moderate bone marrow toxicity coupled with the high costs are a limiting factor [23,24]. Numerous biologic targets, including VEGF-inhibitors, epidermal growth factor receptor inhibitors, poly-ADP-ribose polymerase inhibitors, and anti folate agents have recently been studied to determine their activity in the treatment of EOC [25,26].

Ovarian cancer tumor cells posses VEGF receptors on their surface suggesting a direct anti-tumor effect in addition to an anti-angiogenesis effect. Neutralization of VEGF activity appears to reduce malignant ascites formation [27,28].

Bevacizumab has shown activity in the treatment of EOC, with 15-20% response rate and up to 50% PFS at 6 months in phase II clinical trials in advanced EOC [7-9]. Before routine use of bevacizumab can be recommended in recurrent EOC, however, further analyses of cost, tolerability and optimal biological dose are necessary. A limited number of studies have evaluated the optimal bevacizumab dosage and its correlation with response rate in different tumors. At variance, several clinical trials have assessed dose–response relationships of intravitreal injections of bevacizumab (1.25 mg versus 2.5 mg) for the treatment of diffuse diabetic macular edema and choroidal neo-vascularization associated with age-related macular degeneration [19,20]. In these studies different dosages had similar treatment efficacy, but intravitreal injection of the highest dose was associated with higher rate of adverse events.

In a phase I/II dose-escalation trial of bevacizumab in previously treated metastatic breast cancer the starting dose of 3 mg/kg every other week was chosen based on phase I dose-escalation study in solid tumors [18]. Dose escalation from 3 mg/kg to 20 mg/kg was to occur if no objective responses were observed among 15 patients. Although the highest response rate was seen at 10 mg/kg every other week, the sample size was too small to conclude whether doses higher or lower than 10 mg/kg were more or less effective.

In the current study we assessed the safety and activity of low dose bevacizumab, mostly 5 mg/kg q21 days, in combination with either carboplatin or oral cyclofosfamide or weekly paclitaxel in 15 heavily pre-treated patients with advanced stage EOC. The treatment showed to be active, particularly when considering the lowest 95% confidence limit of tumor response, in a patient population for whom little therapeutic options are available. The treatment was well tolerated with low incidence of proteinuria, hypertension, abdominal pain and bleeding. There were no cases of intestinal perforation or grade 4 toxicities, and treatment was never stopped for toxicity.

The addition of low-dose bevacizumab to chemotherapy was quite active with a best tumor response (CR + RP) as high as 73.4% by CT scan and 67% by PET. The attainment of a tumor response by carboplatin rechallange in all four platinum resistant patients is noticeable and deserves to tested in future studies. Previous studies have shown significant activity of bevacizumab in platinum resistant disease [9-11]. Median progression-free survival was 21 months and median overall survival was 24 months, an observation which is in line with the results obtained by O’Malley et al. with paclitaxel/bevacizumab as compared to weekly paclitaxel alone in a similar population [29]. There was no significant correlation between bevacizumab related adverse events and response rate or between bevacizuamb dose and response rate.

We used ^18^ F-FDG PET/CeCT colonography to detect persistence/recurrence of disease and to assess tumor response using RECIST and PERCIST criteria. ^18^ F-FDG PET/TC is now an essential imaging technique in EOC monitoring and is clearly superior to CeCT especially to identify peritoneal deposits and lymph-nodes metastases [30]. ^18^ F-FDG PET/TC is also able to asses treatment response earlier than conventional imaging as CeCT or MRI and metabolic responders have a longer median overall survival than non responders [31]. The combined use of ^18^ F-FDG PET/CT and CeCT, acquired at the same time in the same dedicated PET/CT scanner has been shown to improve the diagnostic accuracy of the ^18^ F-FDG PET/CT alone [32], especially in case of hostile anatomy of the abdomen and pelvis after surgery [32,33]. However one of the major limitation of this diagnostic tool remains the inability to distinguish small peritoneal disease from non specific bowel uptake [32]. Thus we combined ^18^ F-FDG PET/CeCT with colon distension [21] in order to eliminate aspecific bowel activity and to discover small peritoneal nodules close to intestinal wall [34,35].

Our study has several limitations, including the small sample size and the heterogeneity of chemotherapy regimens. Despite these caveats, our case-series study suggests that the combination of low-dose bevacizumab and chemotherapy is well tolerated and active in heavily pretreated patients with advanced EOC. A comparative trial with conventional chemotherapy is warranted.

## Competing interests

The author declares that they have no competing interests.

## Authors’ contribution

DC, CS, DM, PA, BE, RD, PA, PM, GA, GA, ZS, LR, BM, DCA, All authors read and approved the final manuscript.
